# Quantifying the Influence of Delay in Opinion Transmission of COVID-19 Information Propagation: Modeling Study

**DOI:** 10.2196/25734

**Published:** 2021-02-12

**Authors:** Fulian Yin, Xueying Shao, Meiqi Ji, Jianhong Wu

**Affiliations:** 1 College of Information and Communication Engineering Communication University of China Beijing China; 2 Fields-CQAM Laboratory of Mathematics for Public Health Laboratory for Industrial and Applied Mathematics York University Toronto, ON Canada

**Keywords:** COVID-19, delay transmission, dynamic model, Sina Microblog, social media, communication, online health information, health information, public health, opinion, strategy, model, information transmission, delay, infodemiology, infoveillance

## Abstract

**Background:**

In a fast-evolving public health crisis such as the COVID-19 pandemic, multiple pieces of relevant information can be posted sequentially on a social media platform. The interval between subsequent posting times may have a different impact on the transmission and cross-propagation of the old and new information that results in a different peak value and a final size of forwarding users of the new information, depending on the content correlation and whether the new information is posted during the outbreak or quasi–steady-state phase of the old information.

**Objective:**

This study aims to help in designing effective communication strategies to ensure information is delivered to the maximal number of users.

**Methods:**

We developed and analyzed two classes of susceptible-forwarding-immune information propagation models with delay in transmission to describe the cross-propagation process of relevant information. A total of 28,661 retweets of typical information were posted frequently by each opinion leader related to COVID-19 with high influence (data acquisition up to February 19, 2020). The information was processed into discrete points with a frequency of 10 minutes, and the real data were fitted by the model numerical simulation. Furthermore, the influence of parameters on information dissemination and the design of a publishing strategy were analyzed.

**Results:**

The current epidemic outbreak situation, epidemic prevention, and other related authoritative information cannot be timely and effectively browsed by the public. The ingenious use of information release intervals can effectively enhance the interaction between information and realize the effective diffusion of information. We parameterized our models using real data from Sina Microblog and used the parameterized models to define and evaluate mutual attractiveness indexes, and we used these indexes and parameter sensitivity analyses to inform optimal strategies for new information to be effectively propagated in the microblog. The results of the parameter analysis showed that using different attractiveness indexes as the key parameters can control the information transmission with different release intervals, so it is considered as a key link in the design of an information communication strategy. At the same time, the dynamic process of information was analyzed through index evaluation.

**Conclusions:**

Our model can carry out an accurate numerical simulation of information at different release intervals and achieve a dynamic evaluation of information transmission by constructing an indicator system so as to provide theoretical support and strategic suggestions for government decision making. This study optimizes information posting strategies to maximize communication efforts for delivering key public health messages to the public for better outcomes of public health emergency management.

## Introduction

In the absence of effective treatment or a vaccine, the success or failure of mitigating COVID-19 transmission in the population relies heavily on the effectiveness of social distancing, self-protection, case detection, quarantine, isolation, and testing. The effectiveness of these nonpharmaceutical interventions depends on the active participation and engagement of residents in the community, which is substantially influenced by the public opinion. Given the short disease transmission doubling time, the timing (and hence time lags) in the communication of critical public health information to the community has a profound impact on the outcome of public adherence to nonpharmaceutical measures and, ultimately, on the outcome of outbreak mitigation. Adding to the challenging of effective communications is the cross-propagation of relevant, and sometimes inconsistent, pieces of information that enter social media at different time points. This calls for a strategy of optimizing the timing of posting key information in social media during a fast-evolving pandemic.

**Figure 1:**
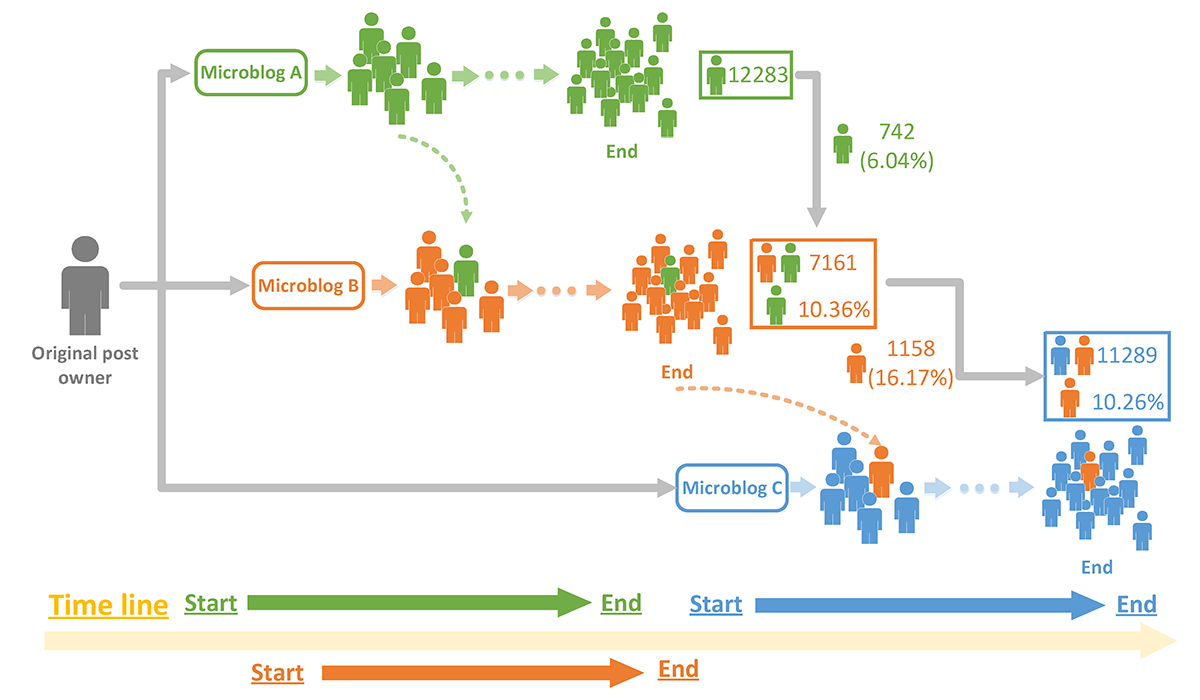
Cross-propagation of three relevant pieces of information related to the COVID-19 pandemic, posted in sequence on Sina Microblog.

Figure 1 shows the cross-propagation of three pieces of related information about COVID-19: titled as “Just want a regular 20200202,” “Announcement of donation acceptance by Wuhan JinYinTan hospital,” and “Three cases of community transmission were reported in Shenzhen for the first time.” These pieces of information were posted in Sina Microblog, with different beginning and ending time points marked in the (horizontal) timeline. Almost immediately after reading information A, some users forwarded information B, so both pieces of information shared similar life cycle but with beginning and ending time points close to each other. There were 12,283 information A users, among which 742 (6.04%) forwarded information B. Eventually, 7161 of users forwarded information B, and those who simultaneously forwarded information A accounted for 10.36% (n=7161). Information C was then released, and users of information B started to forward information C. At the end, among 7161 users of information B, 1158 (16.17%) also forwarded information C, accounting for 10.26% (n=11,289) of the users for C.

In general, relevant information, when posted with an appropriate time lag, can attract the interest of social media users in public hot events by increasing the efficiency of dissemination of a group of information cross-propagated. It is an important topic of research; the main objective of our study is to understand the information cross-propagation dynamics to inform optimal strategies of posting relevant information in an appropriate time sequence to ensure their maximum interaction for effective copromotion during a public health emergency situation.

To the best of our knowledge, no appropriate model framework has been developed and analyzed to examine the impact of information cross-propagation dynamics for a group of relevant information that is posted subsequently. Here, we try to fill this gap by proposing a susceptible-forwarding-immune model with time-delayed posting and transmission. We developed and illustrated this framework, and parametrized our model by using the forwarding quantity that represents public attention to some popular opinions on the COVID-19 pandemic. Our focus is on the dynamic interactions among several pieces of information posted sequentially, and we aim to examine the impact of time lags between different posting time points on the evolution and steady states of cross-propagation.

In the field of information propagation dynamics, there is a strong similarity between the propagation of rumors and a pathogen’s spread in the population [1]. Many studies have used epidemic models to examine rumor propagation in the hope that the negative influence of rumors can be eliminated or at least minimized. For example, the susceptible-infected-exposed-recovered (SIER) model [2-5], susceptible-infected model [6-9], susceptible-infected-susceptible [10,11] model, and susceptible-infected-recovered (SIR) model [12-14] have all been developed and recognized as classical propagation dynamics models.

The development of the internet and the enrichment of social media mandate further extensions of traditional models to reflect novel transmission mechanisms and to take advantage of data from multiple platforms. Gu and Cai [15] and Gu et al [16] proposed the forget-remember mechanism to study the spreading process in a 2-state model. Zhao et al [17] combined the forgetting mechanism and the SIR model to represent the rumor spreading process in an online social blogging platform LiveJournal. In 2014, Zhao et al [18] integrated the refutation mechanism in homogeneous social networks into the SIR model and analyzed the dynamic process of rumor propagation. Considering the three influencing factors of enterprises affected by rumors, pinion leaders, and a microblog platform, an SIR model based on browsing behavior was constructed to explain how rumors spread among followers under the influence of different rumor refuting measures [19]. Other features of the new media were further incorporated by Zhao et al [20]. Borge-Holthoefer et al [21] considered the case when spreaders were not always active and an ignorant was not interested in spreading the rumor, and then separately introduced these ideas into two different models. They concluded that these models provided higher adhesion to real data than classical rumor spreading models. In 2020, Yin et al [22] considered the user’s behavior of re-entering new topics and proposed a multiple-information susceptible-discussing-immune model to investigate COVID-19–relevant information propagation in the Chinese Sina Microblog. Ding et al [23] proposed an improved SIR model, which used differential equations to study the rule of information transmission on media platforms and predicted microblog information accurately. Wang et al [24] proposed a modeling method that considers Weibo propagation behavior based on the susceptible-infected-susceptible model, so the forwarding trend in the future can be predicted. Zhang et al [25] focused on the impact of media transmission and interpersonal relationships on information propagation and then proposed the media and interpersonal relationship susceptible-infected-exposed-recovered model. Zhao et al [26] developed a new rumor spreading model called the susceptible-infected-hibernator-removed model, introducing a new kind of people-hibernators to reduce the maximum rumor influence. Woo et al [27] proposed an event-driven SIR model based on the impact of news releases on social media to reflect the impact of specific events on opinion diffusion. Yao et al [28] concentrated on examining the influence of different interactions among information on the spread of public opinion and modeling based on the SIR model, which verified the otherness of public opinion under a distinct information environment. For other studies relevant to our paper, see [29-32]. In particular, Tanaka et al [32] added a new module to the traditional model by using two data sets from the Japanese Mixi and Facebook rather than a single data set.

Many studies on cross-transmission in disease diffusion are highly significant to study the cross-propagation of information. Feng et al [33] established a mathematical model that incorporated the virus mutation dynamics in the transmission of the Chikungunya virus among mosquitoes and humans. However, the important phenomenon of time lag in posting and cross-propagation of relevant information for information dissemination in real social media networks has not been adequately addressed in these earlier studies. We noted that Zan [34] studied the double rumors spreading with different launch times, in which the new rumor was launched with a certain delay but also could interact with the old rumor. Zan [34] proposed two classes of double-rumors spreading models: a double-susceptible-infected-recovered model, where it was assumed that the rumor was disseminated by direct contacts of infective nodes with others, and a comprehensive double-susceptible-infected-recovered model, with which the authors studied the whole spread situation of all rumors with a focus on determining how many people did not spread all rumors in the entire period or how many were spreading or had spread at least one of the rumors.

In comparison with the aforementioned studies, here we consider the phenomenon where at different propagation stages of a piece of information posted in social media other pieces of information are posted, and their relevance in contexts and posting time sequence combined generate an outbreak for each piece of information and, more importantly, cross-propagation in which users of one piece of information forward other pieces of information they are exposed to later. We developed two classes of dynamic propagation models that focus on the single information transmission and multi-information cross-propagation patterns during explosive and quasi–steady-state periods of the information posted sequentially. We aim to examine emphatically the influence of different participating groups of posted information on the spread of the information from the participating groups. As populations who have forwarded, whether exposed or not exposed to relevant posted information, are attracted to a new piece of information differently, by introducing and analyzing the impact of *attractiveness indexes* on relevant information propagation and examining the significant factors of delaying in posting relevant information propagation, we inform strategies for sequentially posting relevant information to achieve effective communication of key public opinions. We will illustrate this with data from public opinions about COVID-19 pandemic management.

## Methods

### Large Delay in Transmission Susceptible-Forwarding-Immune Dynamics Model

**Figure 2:**
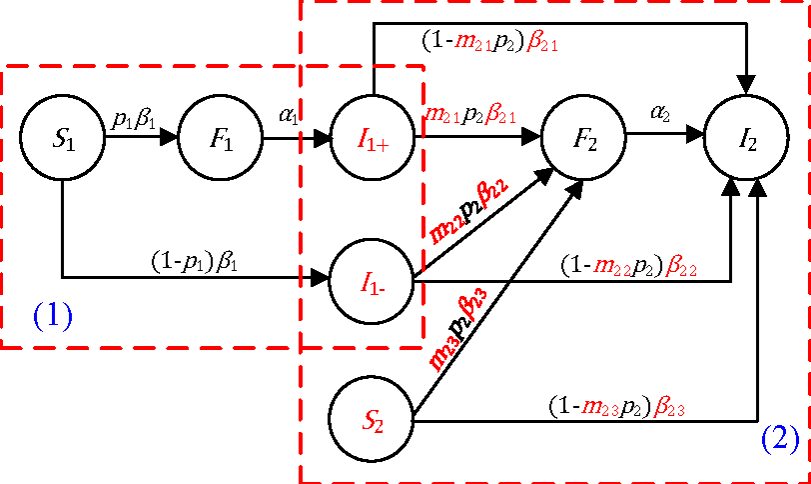
A schematic illustration of the information cross-spreading, where a new piece of information is posted during the quasi steady-state period of an already posted information.

The structure of our proposed large interval delay in transmission susceptible-forwarding-immune (LTI DT-SFI) dynamics model is shown in Figure 2. There are two phases involved. In phase 1, a piece of stand-alone information (information 1) is spreading; and in phase 2, another piece of information (information 2) is posted during the quasi–steady-state period of the posted information.

#### Phase 1: Post a Piece of Stand-alone Information

The propagation dynamics during phase 1 for one piece of posted information is modeled based on the traditional susceptible-forwarding-immune [35] model, with a novel stratification of the immune population. Namely, there will be two classes of immune populations (as far as information 1 is concerned): those who have forwarded the posted information but are no longer in the active period of forwarding this posted information (*I*_1+_) and those who have been exposed to the information but are not interested in forwarding it (*I*_1–_). This distinction of immunity is important, as individuals in these two distinct compartments will have different levels of interest in other relevant information that will be posted later. This will allow us to introduce different measures of attractiveness to new relevant information.

So, in our model, we stratify the population (*N*_1_) into four states: the susceptible state of the posted information (*S*_1_), the forwarding state of the posted information (*F*_1_), the inactive immune state (*I*_1+_), and the direct immune state (*I*_1–_). A susceptible user can be exposed to the posted information with an average exposure rate *β*_1_ and will forward the information with the forwarding probability *p*_1_. The forwarding users can become inactive immune users with an average rate *α*_1_. So a user may have a unique state, with *S*_1_(*t*), *F*_1_(*t*), *I*_1+_(*t*), and *I*_1–_(*t*) denoting the number of users in the susceptible, forwarding, inactive, and direct immune state, respectively. We obtain the following delay in transmission susceptible-forwarding-immune (DT-SFI) dynamics model in phase 1:


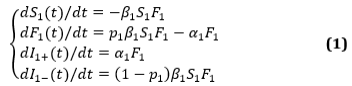


The state transition of different populations in phase 1 can be interpreted as follows: an active forwarding user will contact an average number of *β*_1_*N*_1_ users per unit time, and the probability of a user being a susceptible user of the posted information is *S*_1_(*t*)/*N*_1_, so an active forwarding user will contact *β*_1_*S*_1_(*t*) susceptible users. There are *F*_1_(*t*) active forwarding users of information 1 in total at time t, so *p*_1_*β*_1_*S*_1_(*t*)*F*_1_(*t*) susceptible users will choose to forward the information and become new forwarding users, and (1 – *p*_1_) *β*_1_*S*_1_(*t*)*F*_1_(*t*) will not. As time goes by, *α*_1_*F*_1_(*t*) will go to the immune state from the forwarding period when they do not influence other users as far as information 1 is concerned.

For data fitting purposes, we noted that the Sina Microblog provides important data about any piece of information relevant to COVID-19, the number of cumulative forwarding quantity, given by:


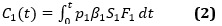


#### Phase 2: Post New Information During the Quasi–Steady-state Period of the Posted Information

Now we consider that a piece of new information is posted at time *t_τ_* when the posted information is already in the quasi–steady state. We introduce the following indexes:

- *An extensive exposure attractiveness index*: for the individuals in the immune state who have forwarded the posted information but are no longer in their active forwarding period. These individuals are more susceptible to the new yet relevant information, as they had interest in the posted information.
- *A mild exposure attractiveness index*: for the direct immune individuals. These individuals have had exposure to the posted information but had shown little interest in the information.
- *An unexposure attractiveness index*: for those who were never exposed to the first posted information

Accordingly, we introduce three states of the population (*N*_2_) for the newly posted information: the susceptible state (*S*_2_), the forwarding state (*F*_2_), and the immune state (*I*_2_). We summarize the notations in Textbox 1.

**Parameter definitions for large delay model.**

**Attractiveness parameters, stratified by the exposure to old information**

- *m*_21_: The extensive exposure attractiveness index that an inactive user of state *I*_1+_ becomes a forwarding user of state *F*_2_.
- *m*_22_: The mild exposure attractiveness index that a direct immune user of state *I*_1–_ becomes a forwarding user of state *F*_2_.
- *m*_23_: The unexposure attractiveness index that a new susceptible user of state *S*_2_ becomes a forwarding user of state *F*_2_.

**Transmission parameters associated with the different attractiveness**

- *β*_21_: The average exposure rate that the inactive users of the old information can contact with the newly posted information.
- *β*_22_: The average exposure rate that the direct immune users of the old information can contact with the newly posted information.
- *β*_23_: The average exposure rate that the new susceptible users can contact with the newly posted information.
- p_2_: The probability that an exposed user will forward the newly posted information.
- *α*_2_: The average rate that a user in the forwarding state of newly posted information becomes inactive to forwarding, where 1/*α*_2_ is the average duration a forwarding user remains active in forwarding newly posted information.

Each user may have a unique state, with *I*_1+_(*t*), *I*_1–_(*t*), *S*_2_(*t*), *F*_2_(*t*), and *I*_2_(*t*) denoting the number of users in the susceptible, forwarding, and immune state, respectively. We obtain the following LTI DT-SFI dynamics model in phase 2:


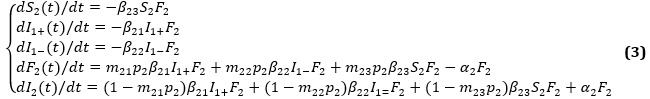


The mass action in phase 2 can be interpreted as follows: an active forwarding user will contact an average number of *β*_21_*N*_2_ inactive immune users of the posted information per unit time, and the probability of a user being an inactive immune user is *I*_1+_(*t*)/*N*_2_, so an active forwarding user will contact *β*_21_*I*_1+_(*t*) inactive immune users, among which *m*_21_*p*_2_*β*_21_*I*_1+_(*t*)*F*_2_(*t*) will choose to forward the new information and (1 – *m*_21_*p*_2_)*β*_21_*I*_1+_(*t*)*F*_2_(*t*) will not, where *F*_2_(*t*) is the number of new active forwarding users at time *t*; an active forwarding user will contact an average number of *β*_22_*N*_2_ direct immune users of the posted information per unit time, and the probability of a user being a direct immune user is *I*_1–_(*t*)/*N*_2_, so an active forwarding user will contact *β*_22_*I*_1–_(*t*) direct immune users, among which *m*_22_*p*_2_*β*_22_*I*_1–_(*t*)*F*_2_(*t*) will choose to forward the new information and (1 – *m*_22_*p*_2_)*β*_22_*I*_1–_(*t*)*F*_2_(*t*) will not, where *F*_2_(*t*) is the number of new active forwarding users at time *t*; an active forwarding user will contact an average number of *β*_23_*N*_2_ susceptible users of the newly posted information per unit time, and the probability of a user being a susceptible user is *S*_2_(*t*)/*N*_2_, so an active forwarding user will contact *β*_23_*S*_2_(*t*) susceptible users, among which *m*_23_*p*_2_*β*_23_*S*_2_(*t*)*F*_2_(*t*) will choose to forward the new information and (1 – *m*_23_*p*_2_)*β*_23_*S*_2_(*t*)*F*_2_(*t*) will not, where *F*_2_(*t*) is the number of new active forwarding users at time *t*.

The forwarding quantity of the newly posted information is given by:


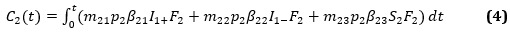


#### The Public Opinion Reproduction Ratio

Since the newly posted Weibo starts at different times and develops differently under the influence of prior information, we defined the information reproduction ratio as:


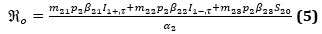


This is the total number of information 2 users generated by introducing a typical information 2 user, at time *τ* after information 1 was posted, during their entire period of active forwarding. The initial population for information 2 has been stratified by the exposure of the entire population to information 1 during the time interval [0, *τ*]. The relative size of 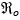 to the unity determines if information 2 can generate an information outbreak; since 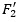 (0) = (*m*_21_*p*_2_*β*_21_*I*_1 + ,τ_ + *m*_22_*p*_2_*β*_22_*I*_1 – ,_*_τ_* + *m*_23_*p*_2_*β*_23_*S*_20_ – *α*_2_) *F*_2_ (0), we concluded that 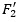 (0)>0 if 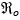
>1.

### Short Delay in Transmission Susceptible-Forwarding-Immune Dynamics Model

**Figure 3:**
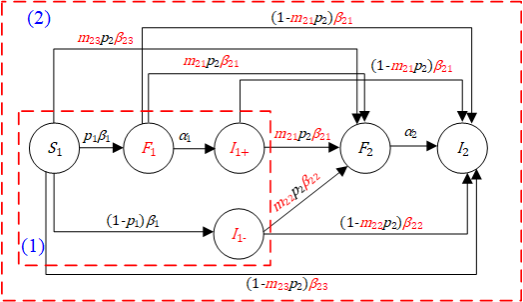
A schematic diagram to illustrate information spreading, when the post time of the newly posted information is during the outbreak period of the posted information.

Our comprehensive short interval delay in transmission susceptible-forwarding-immune (STI DT-SFI) dynamics model based on the forwarding quantity is shown in Figure 3. In this model, we include two phases. In phase 1, a stand-alone piece of information (information 1) is spreading, corresponding to phase 1 in the LTI DT-SFI model. In phase 2, a piece of newly posted information (information 2) is posted at *t_τ_* during an outbreak period of the posted information. Here, we also divide the population into three groups: S population (*S*), F population (*F*_1_, *F*_2_), and I population (*I*_1+_, *I*_1–_, *I*_2_). In particular, we think of the susceptible state of both the posted information and the new information as a whole (*S*). *t_τ_* is the post time of the newly posted information.

#### Phase 1: Post Stand-alone Information

The model is consistent with that of phase 1 of the LTI DT-SFI model.

#### Phase 2: Posting New Information During the Outbreak Period of the Posted Information

Considering that the new information is posted during the outbreak period of the old information, we developed our phase 2 model to describe the concurrent dynamic process of the two related pieces of information. We considered the difference between the population in active forwarding state or the immune state out of the active period and the population in the direct immune state of the first (old) piece of information. Here, we set the following indexes:

- *An extensive exposure attractiveness index*: for the individuals in the forwarding state, who have forwarded the posted information but are still in their active forwarding period, and the individuals in the immune state, who have forwarded the old information but are no longer in their active forwarding period, to indicate that this population will be attracted by the new information due to the relevance of the two pieces of information
- *A mild exposure attractiveness index*: for the direct immune population to portray that the population will be attracted due to a moderate contact
- *An unexposure attractiveness index*: for the integrated susceptible population to describe that the population will be attracted when they have never read the related information

Assuming that the number of users (*N*_3_) who can contact the information in the process of information propagation on Sina Microblog remains unchanged, we introduced three states of the population of the newly posted information: the susceptible state (*S*_1_) that includes the users who can be exposed to the old information and the new information, the forwarding state of the new information (*F*_2_), and the immune state (*I*_2_). The parameters are shown in Textbox 2.

**Parameters definitions for short delay model.**

**Attractiveness parameters, stratified by the exposure to old information**

- *m*_21_: The extensive exposure attractiveness index that an inactive user of state *I*_1+_ becomes a forwarding user of state *F*_2_.
- *m*_22_: The mild exposure attractiveness index that a direct immune user of state *I*_1–_ becomes a forwarding user of state *F*_2_.
- *m*_23_: The unexposure attractiveness index that a new susceptible user of state *S*_2_ becomes a forwarding user of state *F*_2_.

**Transmission parameters associated with the different attractiveness**

- *β*_1_: The average exposure rate that the susceptible users can contact the first information.
- *β*_21_: The average exposure rate that the inactive users of the old information can contact the newly posted information.
- *β*_22_: The average exposure rate that the direct immune users of the old information can contact the newly posted information.
- *β*_23_: The average exposure rate that the new susceptible users can contact the newly posted information.
- *p*_1_: The probability that the susceptible users will forward the first information.
- *p*_2_: The probability that the exposed users will forward the newly posted information.
- *α*_2_: The average rate that a user in the forwarding state of newly posted information becomes inactive to forwarding, where 1/*α*_2_ is the average duration a forwarding user remains active in forwarding newly posted information.

Each user may have a unique state, with *S*_1_(*t*), *F*_1_(*t*), *I*_1+_(*t*), *I*_1–_(*t*), *F*_2_(*t*), and *I*_2_(*t*) denoting the number of users in the susceptible, forwarding, and immune state when *t*>0, respectively. We obtain the following STI DT-SFI dynamics model in phase 2:


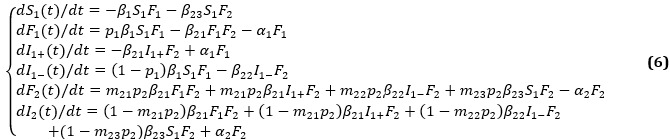


The mass action in phase 2 can be interpreted as follows: an active forwarding user will contact an average number of *β*_21_*N*_3_ inactive immune users and forwarding users of posted information per unit time, and the probability of a user being an inactive immune user and a forwarding user are *I*_1+_(*t*)/*N*_3_ and *F*_1_(*t*)/*N*_3_, respectively, so an active forwarding user will contact *β*_21_*I*_1+_(*t*) inactive immune users and *β*_21_*F*_1_(*t*) forwarding users, among which *m*_21_*p*_2_*β*_21_*I*_1+_(*t*)*F*_2_(*t*) and *m*_21_*p*_2_*β*_21_*F*_1_(*t*)*F*_2_(*t*) will choose to forward the new information, however, (1 – *m*_21_*p*_2_)*β*_2_*I*_1+_(*t*)*F*_2_(*t*) and (1 – *m*_21_*p*_2_)*β*_21_*I*_1_(*t*)*F*_2_(*t*) will not, where *F*_2_(*t*) is the number of new active forwarding users at time *t*. Here, the individuals in the forwarding state and the individuals in the immune state have the same familiarity with the topic content who have forwarded information 1; an active forwarding user will contact an average number of *β*_22_*N*_3_ direct immune users of posted information per unit time, and the probability of a user being a direct immune user is *I*_1–_(*t*)/*N*_3_, so an active forwarding user will contact *β*_22_*I*_1–_(*t*) direct immune users, among which *m*_22_*p*_2_*β*_22_*I*_1–_(*t*)*F*_2_(*t*) will choose to forward the new information and (1 – *m*_22_*p*_2_)*β*_22_*I*_1–_(*t*)*F*_2_(*t*) will not, where *F*_2_(*t*) is the number of new active forwarding users at time *t*; an active forwarding user will contact an average number of *β*_23_*N*_3_ susceptible users, and the probability of a user being a susceptible user is *S*_1_(*t*)/*N*_3_, so an active forwarding user will contact *β*_23_*S*_1_(*t*) susceptible users, among which *m*_23_*p*_2_*β*_23_*S*_1_(*t*)*F*_2_(*t*) will choose to forward the new information and (1 – *m*_23_*p*_2_)*β*_23_*S*_1_(*t*)*F*_2_(*t*) will not, where *F*_2_(*t*) is the number of new active forwarding users at time *t*.

The forwarding quantity of the newly posted information is given by:


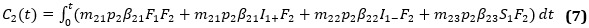


#### The Public Opinion Reproduction Ratio

Considering the initial condition in phase 2, we can obtain the following public opinion reproduction ratio 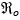. The new information entered at time *τ* during an outbreak period of the posted information, and 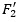 (0) = (*m*_21_*p*_2_*β*_21_*F*_1_*_τ_* + *m*_21_*p*_2_*β*_21_*I*_1 + ,_*_τ_* + *m*_22_*p*_2_*β*_22_*I*_1 – ,_*_τ_* + *m*_23_*p*_2_*β*_23_*S*_1_*_τ_* – *α*_2_)*F*_2_(0). The population will never take off if 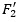 (0) = (*m*_21_*p*_2_*β*_21_*F*_1_*_τ_* + *m*_21_*p*_2_*β*_21_*I*_1 + ,_*_τ_* + *m*_22_*p*_2_*β*_22_*I*_1 – ,_*_τ_* + *m*_23_*p*_2_*β*_23_*S*_1_*_τ_* – *α*_2_)*F*_2_(0)<0 due to the decrease of *S*_10_*_τ_*. It is therefore natural to introduce the following as the STI DT-SFI reproduction ratio:


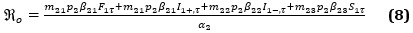


In the same way, the 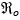 of the STI DT-SFI model denotes the comprehensive public opinion generated by the newly posted Weibo starting at the outbreak period of the posted information. When the reproduction ratio <1, it means that the new public opinion will decline. When the reproduction ratio >1, it indicates that the new public opinion will initially grow exponentially.

### Statistical Analysis

#### Data Description

**Figure 4:**
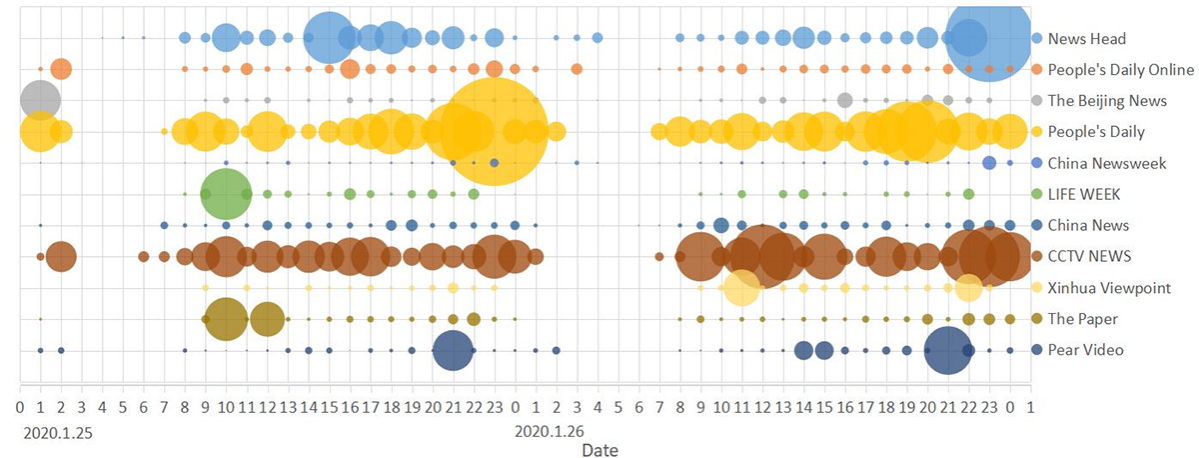
A bubble chart of forwarding quantity of public opinions on COVID-19 information by top ten opinion leaders in Weibos, during an early period of COVID-19 outbreak in China.

Since the COVID-19 outbreak in China, intensive information that were clearly relevant to each other have been frequently posted. Figure 4 shows the total forwarding quantities of Weibos during each 1-hour time frame on January 25, 2020, and January 26, 2020 (data acquisition up to February 19, 2020), for the top 10 opinion leaders of this outbreak event in the Chinese Sina Microblog. As illustrated, those pieces of information with high influence were posted frequently by each opinion leader. At the same time, there was a strong correlation among a series of Weibos posted by these opinion leaders during certain periods. Of all the data shown in Figure 4, People’s Daily issued five Weibos in 1 hour from 10 PM to 11 PM on January 25, and they were forwarded by a total of more than 300,000 users. Therefore, the frequent release of relevant information by original post owners was a common phenomenon in the COVID-19 information propagation, and understanding its effectiveness is important.

**Figure 5:**
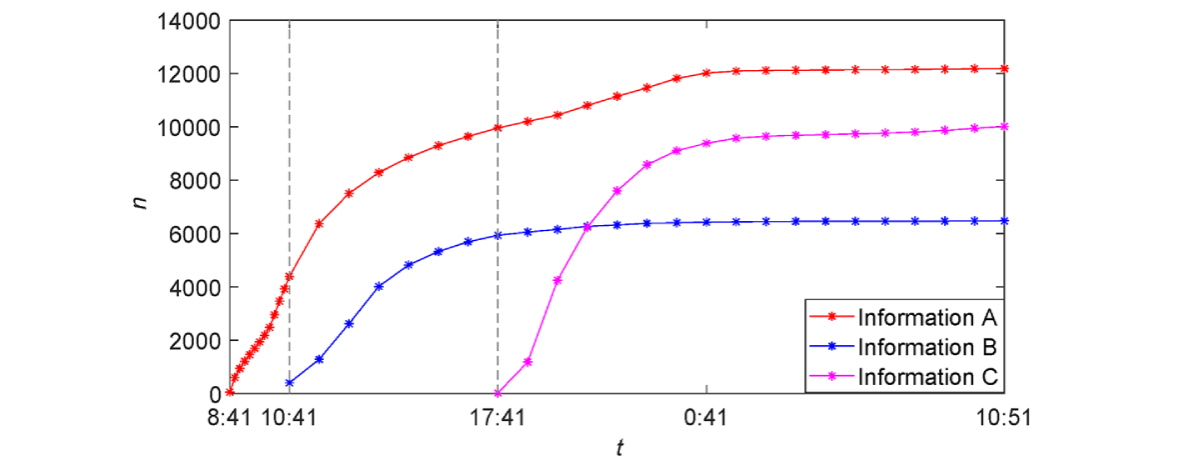
Cumulative forwarding quantity of three pieces of information.

Figure 5 shows the trend of cumulative forwarding users for the three pieces of information in Tables 1-3. It shows that when information A broke out, information B was posted almost immediately. Compared with the information, the outbreak period of information B was shorter and the trend was flatter. Information C was released during the quasi–steady-state period of information B. In comparison with this information, the outbreak period of information C lasted longer; meanwhile, the cumulative forwarding quantity was also larger.

Sequentially releasing two related pieces of information by the same original post owners within the same COVID-19 theme was a common phenomenon. Importantly, different entering times of new information during the spreading process of an old (previously posted) information exhibited different promoting effects on the cross-propagation and cross-promotion of relevant public opinions. Here we focus on users who have been exposed to one posted information that may have a special interest in, and hence are susceptible to, new and relevant information. This represents a remarkable difference from the spread of rumors and other traditional public hot events. Our information cross-propagation DT-SFI models, including the STI DT-SFI dynamics model and the LTI DT-SFI dynamics model, were developed to take into consideration the situations when the relevant information is posted during the outbreak period or during the quasi–steady-state period of the previously posted (old) information.

**Table 1:** Cumulative forwarding quantity of information A posted at 8:41 AM on February 2, 2020.

Time	Information A forwarding quantity, n
0 min	47
10 min	597
20 min	940
30 min	1208
40 min	1458
50 min	1691
60 min	1937
70 min	2182
80 min	2477
90 min	2952
100 min	3461
110 min	3917
2 h	4390
3 h	6366
4 h	7501
5 h	8281
6 h	8846
7 h	9293
8 h	9638
9 h	9954
10 h	10,199
11 h	10,435
12 h	10,795
13 h	11,138
14 h	11,459
15 h	11,812
16 h	12,013
17 h	12,088
18 h	12,109
19 h	12,119
20 h	12,128
21 h	12,136
22 h	12,140
23 h	12,146
24 h	12,157
25 h	12,171
26 h	12,184

**Table 2:** Cumulative forwarding quantity of information B posted at 10:41 AM on February 2, 2020.

Time (hours)	Information B forwarding quantity, n
2	15
3	1281
4	2615
5	4013
6	4817
7	5322
8	5685
9	5932
10	6052
11	6152
12	6264
13	6317
14	6380
15	6401
16	6423
17	6434
18	6447
19	6454
20	6455
21	6456
22	6458
23	6460
24	6461
25	6465
26	6471

**Table 3:** Cumulative forwarding quantity of information C posted at 5:51 PM on February 2, 2020.

Time (hours)	Information C forwarding quantity, n
9	20
10	1180
11	4244
12	6235
13	7595
14	8572
15	9103
16	9381
17	9569
18	9642
19	9680
20	9700
21	9736
22	9764
23	9800
24	9864
25	9943
26	10,006

#### Data Fitting for the LTI DT-SFI Model

#### Parameter Estimation

To fit our model with real data from the Sina Microblog, we used the least squares (LS) method to estimate the LTI DT-SFI model parameters and the initial data. In phase 1, the parameter vector is set as Θ_1_ = (*p*_1_, *β*_1_, *α*_1_, *S*_10_), and the corresponding numerical calculation based on the parameter vector for *C*_1_(*t*) is denoted by 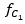 (*k*, Θ_1_). The follow LS error function was used in our calculation:


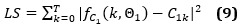


where *C*_1_*_k_* denotes the actual cumulative forwarding populations of the posted information. Similarly, in phase 2, the vector is set as Θ_2_ = (*β*_21_, *β*_22_, *β*_23_, *m*_21_, *m*_22_, *m*_23_, *p*_2_, *α*_2_, *S*_20_), and the corresponding numerical calculation based on the parameter vector for *C*_2_(*t*) is denoted by 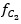 (*k*, Θ_2_). The following LS error function was used in our calculation:


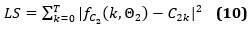


where *C*_2_*_k_* denotes the actual cumulative forwarding populations of the newly posted information. Here, *n*=1, 2 ... represents the different phases, and *k*=0, 1, 2, ... is the sampling time *n*=1, 2, 3. We estimated the parameters of our *LTI DT-SFI* model with the data of information B and information C.

**Figure 6:**
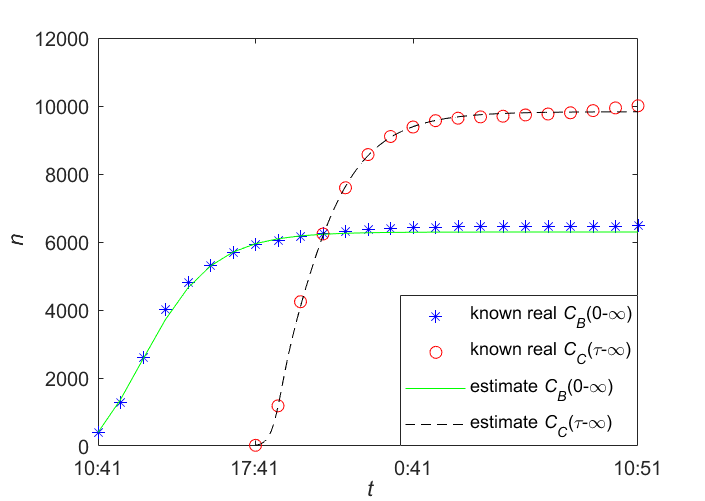
The data fitting results of Information B and Information C.

Figure 6 reports our data fitting results for information B and information C on the real data given in Tables 2 and 3, where the blue star denotes the actual cumulative number of forwarding users of information B; the red star denotes the actual cumulative number of forwarding users of information C; and the green line and the black line denote the estimated cumulative number of forwarding users of information B and information C, respectively.

Tables 4 and 5 give estimated values of important parameters for information B and information C, respectively. We can see in phase 2, when information C was posted during the quasi–steady-state period of information B, the average exposure rate *β*_21_ was the largest, indicating that an inactive user of the posted information B was more susceptible to the newly posted information C; the average exposure rate *β*_23_ was small, indicating that a susceptible user of the newly posted information C contacted the information at a lower rate. In addition, among the three attractiveness indexes, the index *m*_22_ is the largest, which indicates that information C had the strongest appeal to a direct immune user of information B and has the least attractiveness to an inactive user of the posted information B.

By comparison, there is a difference between the initial time of a new piece of information at the outbreak phase and at the quasi–steady-state phase of the posted information. When the initial time of new information is in the outbreak phase of the posted information, the value of the average immune rate *α*_2_ is generally higher than the value in the quasi–steady-state phase, which is due to the rapid outbreak of information (a large amount of information updates and iterations). The average active duration 1/*α*_2_ of forwarding users of the new piece of information where users can influence other users to contact information is shorter. Similarly, the average forwarding probability *p*_2_ of the initial time in the outbreak period is also higher than the value in the quasi–steady-state phase, which conforms to the fact that people are more willing to participate in the discussion successively when exposed to relevant information in the short term. In comparison, the value of average contact rate *β*_21_ in the outbreak phase is lower than that in the quasi–steady-state phase, indicating that the population who has forwarded information will be larger based on a relatively larger contact population. *β*_22_ and *β*_23_ of the initial time in the outbreak period are higher than those of the initial time in the quasi–steady-state phase, indicating that continuous exposure to relevant information in the short term (when the initial time of the new piece of information is in the outbreak phase of a posted information) would attract people who had not participated in the new transmission to forward and spread the information. All average attractiveness indexes of the initial time in the quasi–steady-state phase are larger, which indicates that information that is re-exposed to users after a period of time will inspire their freshness and make them pay more attention to the information itself.

**Table 4:** Values of some important parameters, estimated for information B.

Parameter	Estimated value	Standard error	Minimum	Maximum
*S*_10_	5.6458 × 10^6^	81.0304	0.0000	1.0000 × 10^8^
*α*_1_	1.5757	0.0427	0.0000	4.0000
*β*_1_	1.7901 × 10^–4^	1.7463 × 10^–5^	0.0000	1.0000
*p*_1_	0.0020	1.5023 × 10^–4^	0.0000	1.0000

**Table 5:** Values of important parameters, estimated for information C.

Parameter	Estimated value	Standard error	Minimum	Maximum
*S*_20_	7.4439 × 10^6^	1.3770 × 10^3^	0.0000	1.0000 × 10^8^
*α*_2_	0.9858	0.0993	0.0000	4.0000
*β*_21_	0.8994	0.2829	0.0000	1.0000
*β*_22_	0.0023	0.0023	0.0000	1.0000
*β*_23_	1.0871 × 10^–4^	1.2088 × 10^–4^	0.0000	1.0000
*m*_21_	0.2468	0.1132	0.0000	2.0000
*m*_22_	1.9895	0.1087	0.0000	2.0000
*m*_23_	0.5559	0.2646	0.0000	2.0000
*p*_2_	2.9516 × 10^–4^	1.8597 × 10^–4^	0.0000	1.0000

#### Data Fitting for the STI DT-SFI Model

#### Parameter Estimation

To use our model to explore some distinctions of the qualitative behaviors for prediction, we used the LS method to estimate the STI DT-SFI model parameters and the initial data of our model. The vector is set as Θ_3_ = (*p*_1_, *β*_1_, *α*_1_, *p*_2_, *β*_21_, *β*_22_, *β*_21_, *m*_21_, *m*_22_, *m*_23_, *α*_2_, *S*_10_), and the corresponding numerical calculation based on the parameter vectors for *C*_1_(*t*) and *C*_2_(*t*) are denoted by 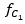 (*k*, Θ_3_) and 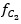 (*k*, Θ_3_), respectively. The following LS error function was used in our calculation:


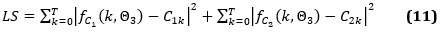


where *C*_1_*_k_* and *C*_2k_ denote the actual cumulative forwarding populations of the posted information and the newly posted information; here, *n*=1, 2, 3 represents the different phases, and *k*=0, 1, 2, ... is the sampling time *n*=1, 2, 3. We estimated the parameters of our *STI DT-SFI* model with the data of information A and information B.

**Figure 7:**
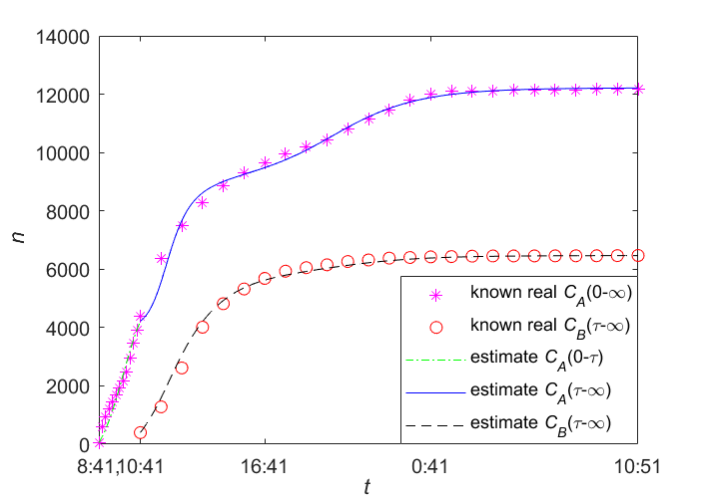
The data fitting results of Information A and Information B.

In the data fitting of the *STI DT-SFI* model, we used the same method as for the LTI DT-SFI model to fit the data of information A and information B. As shown in Figure 7, we performed data fitting of information A and information B on the real data in Tables 1 and 2, where the pink star denotes the actual cumulative number of forwarding users of information A; the red star denotes the actual cumulative number of forwarding users of information B; the green line and the blue line denote the estimated cumulative number of forwarding users in the early and later period of information A, respectively; and the black line denotes the estimated cumulative number of forwarding users of information B. It can be seen that our STI DT-SFI model achieves accurate estimation.

Table 6 gives some important values of parameter (relevant to the early period of the outbreak) estimation for information A, and Table 7 gives some important values of parameter estimation for the later period data of information A and all data of information B. We can see in phase 2, when information B was posted during the outbreak period of information A, the average exposure rate *β*_21_ and *β*_22_ are much larger than *β*_1_ and *β*_23_, which indicates that users who have been exposed to information A will contact information B at a greater rate than new susceptible users. In addition, the unexposure attractiveness index *m*_23_ is the largest among the three attractiveness indexes since the time interval between two information posts is small, and people who have not been exposed to relevant information may have a greater interest in new information; the outbreak of information B has the strongest appeal to susceptible users.

**Table 6:** Values of some important parameters, estimated for information A.

Parameters	Estimated value	Standard error	Minimum	Maximum
*S*_10_	5.1682 × 10^4^	28.3841	0.0000	1.0000 × 10^7^
*α*_1_	3.9986	0.4214	0.0000	4.0000
*β*_1_	8.2700 × 10^–5^	1.5673 × 10^–5^	0.0000	1.0000
*p*_1_	0.9823	0.1543	0.0000	1.0000

**Table 7:** Values of important parameters, estimated for information B.

Parameter	Estimated value	Standard error	Minimum	Maximum
*S*_10_	2.1494 × 10^6^	208.4607	1.0000 × 10^5^	1.0000 × 10^7^
*α*_1_	3.4777	0.1360	2.5000	3.50000
*α*_1_	1.9159	0.0706	1.5000	3.50000
*β*_1_	3.6601 × 10^–4^	1.1663 × 10^–4^	0.0000	4.0000 × 10^–4^
*β*_21_	0.0037	7.4426 × 10^–4^	0.0000	0.0040
*β*_22_	0.8184	0.0932	0.0000	1.0000
*β*_23_	6.8834 × 10^–5^	3.6532 × 10^–5^	0.0000	1.00007.4426 × 10^–4^
*m*_21_	0.0406	0.0196	0.0000	0.2000
*m*_22_	0.0109	0.0093	0.0000	0.2000
*m*_23_	0.1868	0.0445	0.0000	0.2000
*p*_1_	0.0091	0.0026	0.0000	0.0200
*p*_2_	0.0788	0.0346	0.0000	0.2000

## Results

### Influencing Factors Analytics: Information Release and Dissemination for the LTI DT-SFI Model

**Figure 8:**
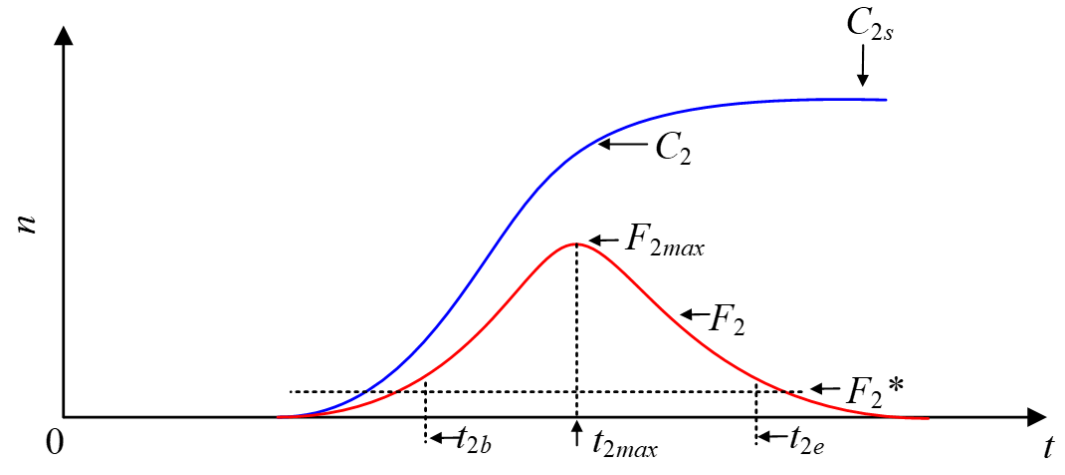
Some summative indices of a newly posted information that cross-propagating with an old information.

To make a qualitative and quantitative analysis of the delay in transmission, we introduced some additional indexes, shown in Figure 8, and show how these can be used to characterize the cross-propagation. We considered different effects of the posted information on newly posted information when the posted information has reached a quasi–steady state or is still in its outbreak period.

- *The outbreak peak *F*_2max_*: the maximum of curve *F*_2_, which reflects the peak user values of the newly posted information
- *The final size C_2S_*: the stable state of curve *C*_2_, which gives the final size of the total number of users of the newly posted information
- *The outbreak time t_2b_, the end time t_2e_, and the duration t_2i_*: the definition depends on the outbreak threshold *F*_2_* set in advance so that *F*_2_(*t*_2b_) = *F*_2_*=*F*_2_(*t*_2e_). Here, *t*_2b_ denotes the outbreak time of the newly posted information, *t*_2e_ denotes the end time, and *t*_2i_ = *t*_2e_–*t*_2b_ denotes the duration of the newly posted information transmission. These time indexes will help us judge the start and end of the newly posted information transmission.
- *The outbreak velocity V_2o_ and the declining velocity V_2d_*: the definition depends on *V*_2o_= (*F*_2max_–*F*_2_*) / (*t*_2max_–*t*_2b_) and *V*_d_= (*F*_2max_–*F*_2_*) / (*t*_2e_–*t*_2max_) when *F*_2_(*t*)=*F*_2max_ and *t*_2max_ is definite, which reflects the speed of the outbreak and the decline of the newly posted information.

**Figure 9:**
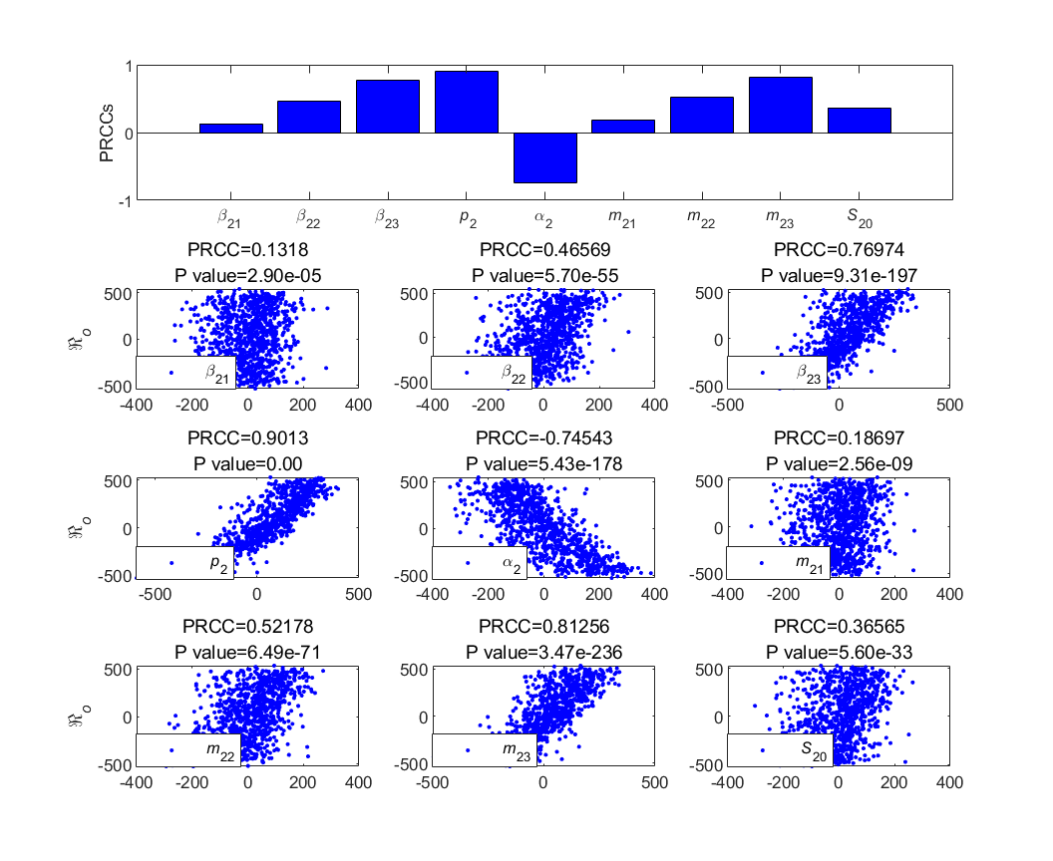
PRCC results and PRCC scatterplots with indexes 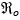 for different parameters of the newly posted information in the large interval delay in transmission susceptible-forwarding-immune model. PRCC: partial rank correlation coefficient.

**Figure 10:**
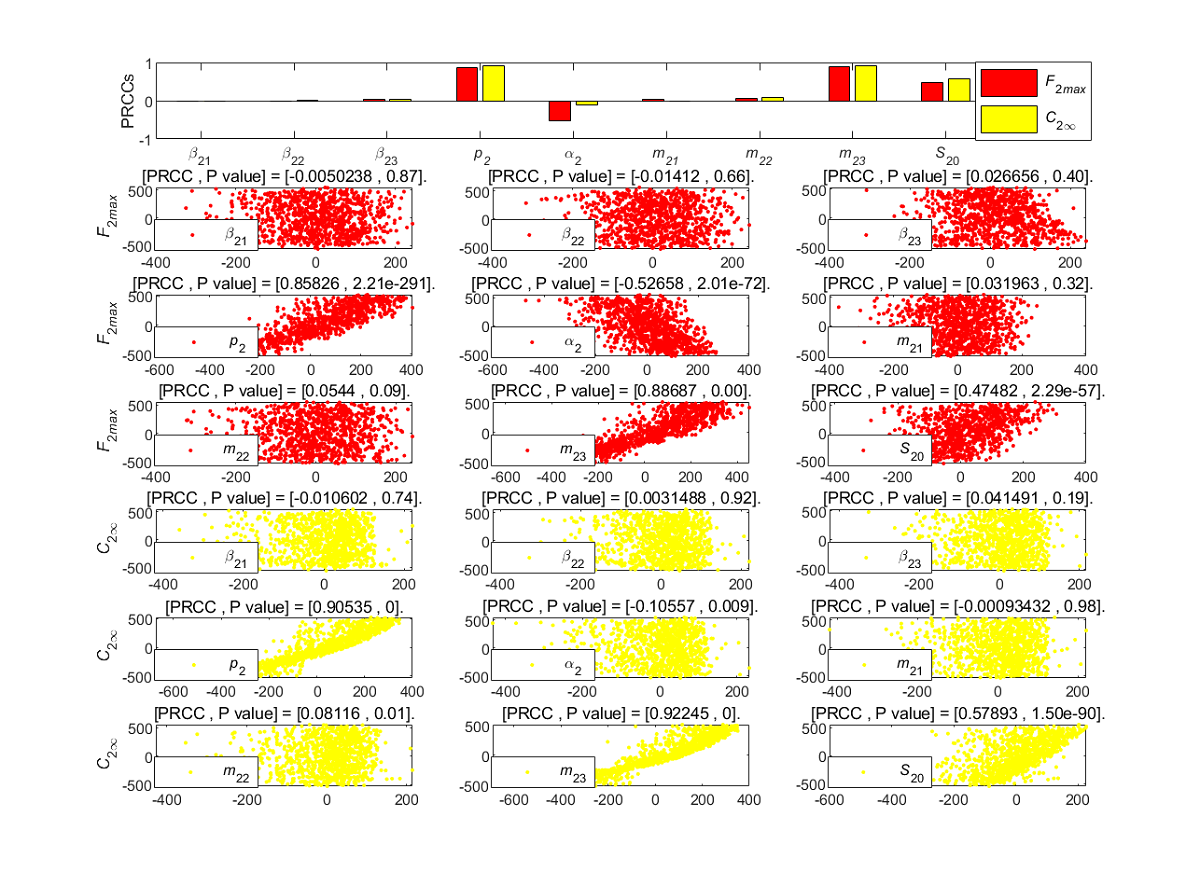
PRCC results and PRCC scatterplots with indexes *F*_2*max*_ and *C*_2∞_ for different parameters of the newly posted information in the large interval delay in transmission susceptible-forwarding-immune model. PRCC: partial rank correlation coefficient.

**Figure 11:**
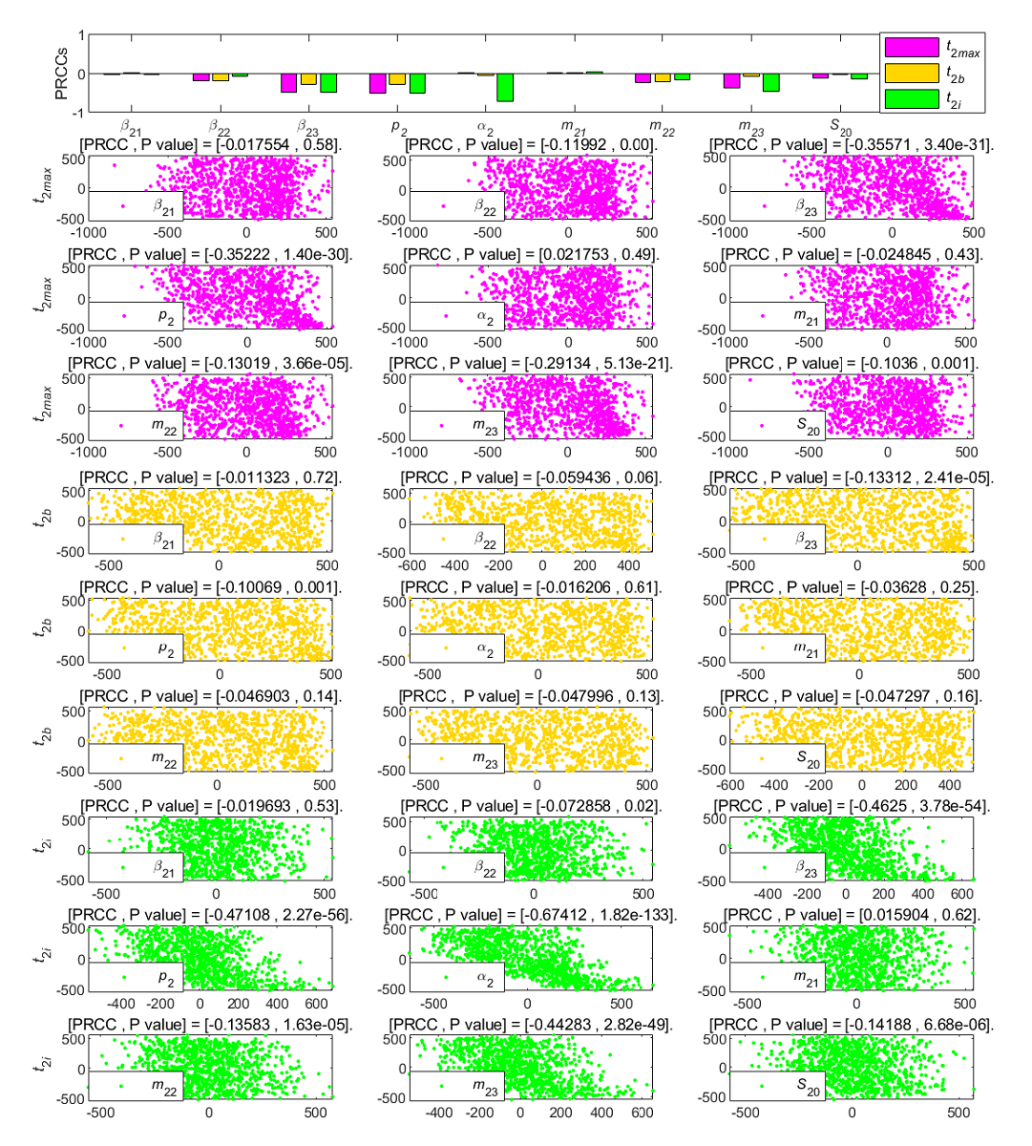
PRCC results and PRCC scatterplots with indexes *t*_2*max*_, *t*_2*b*_, and *t*_2*i*_ for different parameters of the newly posted information in the large interval delay in transmission susceptible-forwarding-immune model. PRCC: partial rank correlation coefficient.

**Figure 12:**
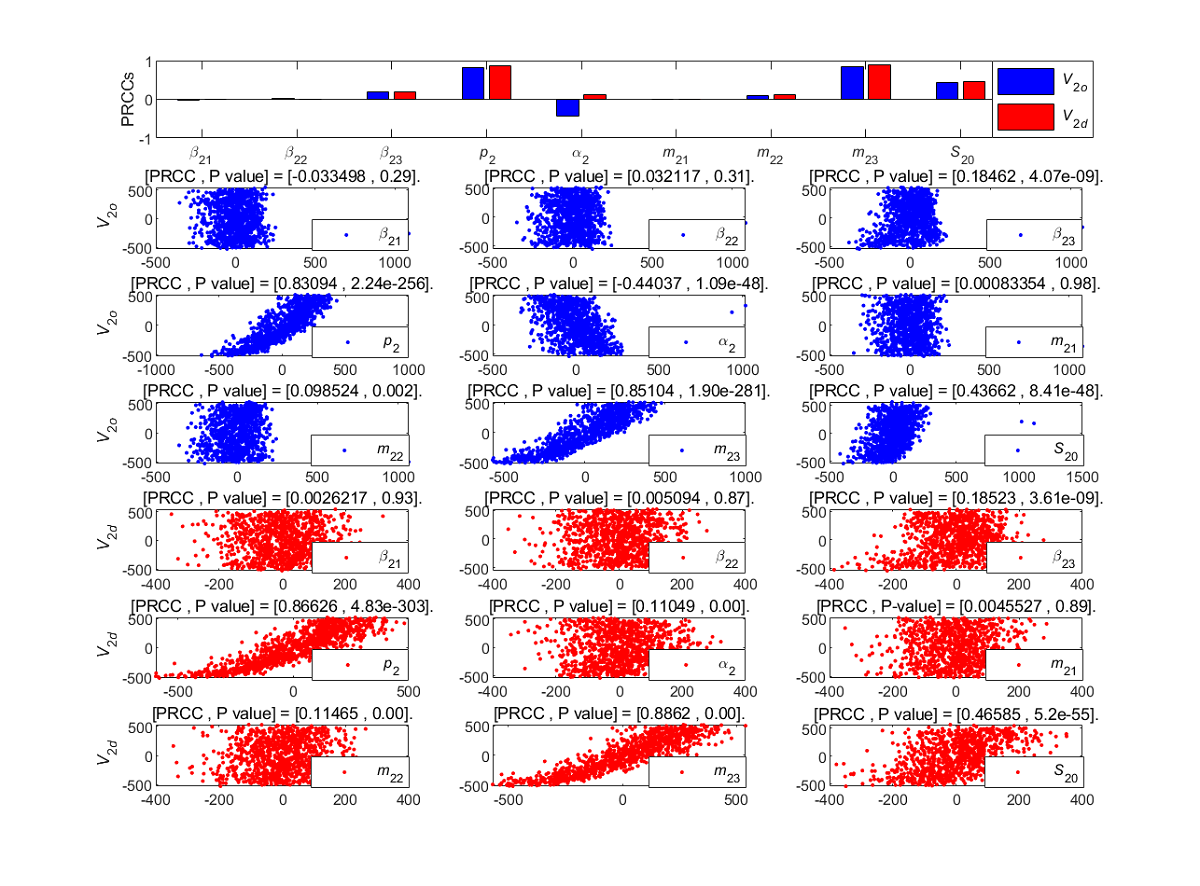
PRCC results and PRCC scatterplots with indexes *V*_2*o*_ and *V*_2*d*_ for different parameters of the newly posted information in the large interval delay in transmission susceptible-forwarding-immune model. PRCC: partial rank correlation coefficient.

To further analyze the different parameters responsible for the LTI DT-SFI model, we performed an analysis of partial rank correlation coefficients [36] to evaluate the sensitivity based on 1000 samples for various input parameters against the threshold condition. According to the histogram and scatter diagram of 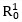 dependence, when the correlation is positive, it means that, with the increase of the value of the parameter, the corresponding index value will increase; on the contrary, when the correlation is negative, the index will decrease as the parameter decreases. Figures 9-12 give the partial rank correlation coefficient results and partial rank correlation coefficient scatterplots with indexes 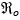, *F*_2_*_max_*, *C*_2∞_, *t*_2_*_b_*, *t*_2_*_i_*, *t*_2_*_max_*, *V*_2_*_o_*, and *V*_2_*_d_* with nine parameters (*β*_21_, *β*_22_, *β*_23_, *p*_2_, *α*_2_, *m*_21_, *m*_22_, *m*_23_, and *S*_20_) of the newly posted information in the LTI DT-SFI model, respectively.

Figure 9 shows the effect of parameters on the public opinion reproduction ratio 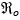 of the delay in transmission in the LTI DT-SFI model. 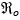 was strongly positively affected by the average exposure rate *β*_22_, the mild exposure attractiveness index *m*_22_, the average exposure rate *β*_23_, the unexposure attractiveness index *m*_23_, and the forwarding probability *p*_2_, and strongly negatively affected by the average immune rate *α*_2_. The positive correlation effects of the parameters *β*_21_ and *m*_21_ were relatively weak. Overall, strategies to increase the parameters *β*_22_, *β*_23_, *p*_2_, *m*_22_, *m*_23_, and initial value *S*_20_ or to decrease the *α*_2_ can enhance the transmission capability of the newly posted information.

From Figure 10, the parameters have a similar effect on the forwarding peak *F*_2_*_max_* and the cumulative forwarding population *C*_2∞_. The unexposure attractiveness index *m*_23_, the forwarding probability *p*_2_, and the initial value *S*_20_ of susceptible individuals have a decisive positive influence on the forwarding peak value *F*_2_*_max_* and the final size *C*_2∞_ of delayed information propagation. The effects of the extensive and mild exposure attractiveness parameters that portray the participation of the population of the posted information were very weak. The aforementioned results indicate that the time interval is long between the two delays in transmission information since the new information was posted in a quasi–steady state into the propagation; at this time, most individuals who have been exposed to the posted information have entered the immune state. In addition, most individuals will no longer care about the relevant content due to the possibility of forgetting or leaving the social network platform. The aforementioned conclusions show that when the posted information enters the steady state, the effect of the individuals who have contacted the posted information is not obvious. Therefore, the information transmission can be promoted by influencing the number of the new susceptible population.

Figure 11 shows the effect of parameters on the climax time *t*_2_*_max_*, the outbreak time *t*_2_*_b_*, and the duration *t*_2_*_i_* of the delay in transmission. After mastering the influencing factors of *t*_2_*_b_* and *t*_2_*_i_*, the end time of transmission *t*_2_*_e_* can be calculated. The climax time *t*_2_*_max_*, the outbreak time *t*_2_*_b_*, and the duration *t*_2_*_i_* are negatively affected by parameters *β*_23_, *p*_2_, and *m*_23_ in the same way. In comparison, these parameters have the least impact on *t*_2_*_b_*, especially *m*_23_. The parameter *m*_22_ had a weak negative correlation effect on each time index, and the parameter *α*_2_ was the main factor to control the duration *t*_2_*_i_*, which plays a strong negative correlation effect.

From Figure 12, the unexposure attractiveness index *m*_23_ and the forwarding probability *p*_2_ had major positive correlation effects on the outbreak velocity *V*_2_*_o_* and the declining velocity *V*_2_*_d_*, and the initial value *S*_20_ of susceptible individuals had a mild positive effect on these two indexes. Moreover, the parameter *α*_2_ had a strong negative effect on the *V*_2_*_o_*. In addition, the effects of other parameters on the velocities were not important. That is to say, the *V*_2_*_o_* and *V*_2_*_d_* will increase accordingly when the parameters *m*_23_, *p*_2_, and initial value *S*_20_ increase. At the same time, the *V*_2_*_o_* increases with the reduction of parameter *α*_2_. By contrast, the effect of *m*_23_ on the velocities was greater.

**Figure 13:**
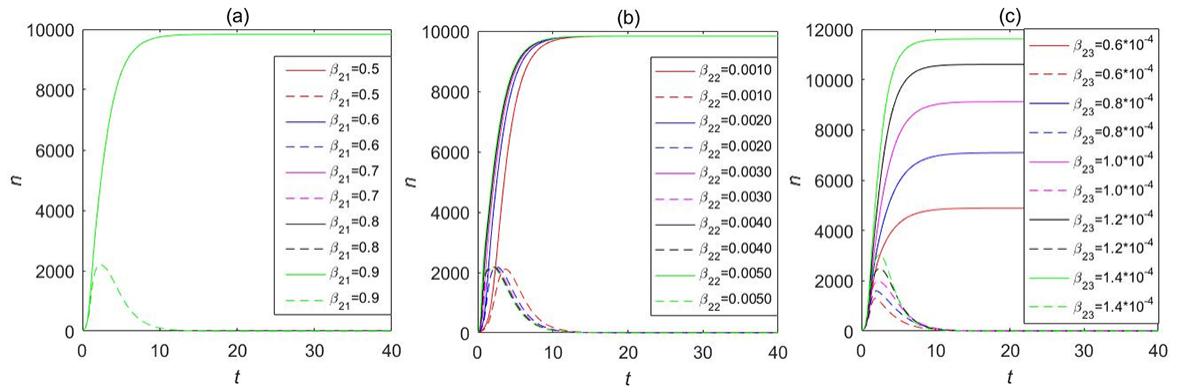
The influence of the average exposure rates on the instantaneous forwarding population *F*_2_(*t*) and the cumulative forwarding population *C*_2_(*t*) in the large interval delay in transmission susceptible-forwarding-immune model.

**Figure 14:**
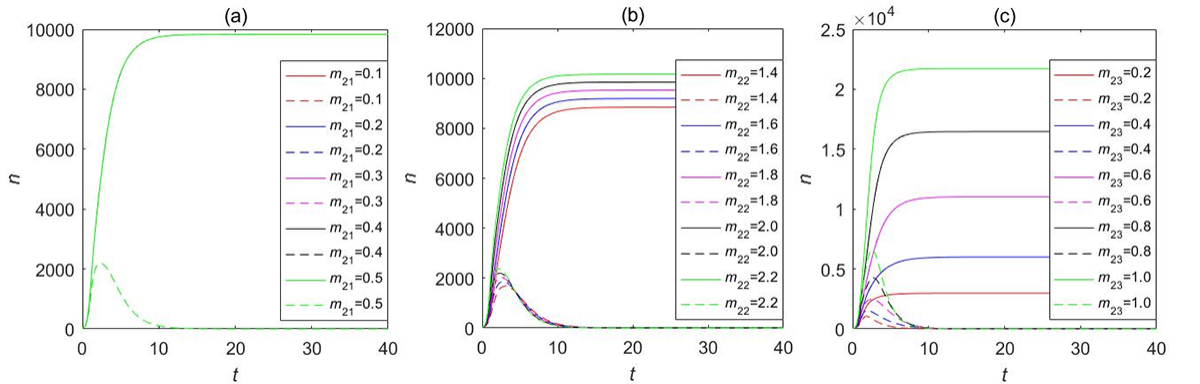
The influence of the attractiveness indexes on the instantaneous forwarding population *F*_2_(*t*) and the cumulative forwarding population *C*_2_(*t*) in the large interval delay in transmission susceptible-forwarding-immune model.

Our LTI DT-SFI model concentrates on the influence of the average exposure rates and attractiveness indexes on the instantaneous forwarding population *F*_2_(*t*) and the cumulative forwarding population *C*_2_(*t*) as shown in Figures 13 and 14, respectively, and the variation of parameters over time determines the propagation indexes. By comparing and analyzing the influence of average contact rates and attractiveness indexes in Figures 11 and 12 with the variation of one parameter while fixing other parameters, *β*_23_ and *m*_23_ have a similar overall trend of the effects on the instantaneous forwarding population *F*_2_(*t*) and the cumulative forwarding population *C*_2_(*t*) of the new information. With the increase of the parameters of *β*_23_ and *m*_23_, the outbreak will accelerate, the instantaneous number of individuals in the forwarding state can reach a higher peak, and the final size will be larger. In addition, the average exposure rate of *β*_22_ and the mild exposure attractiveness index *m*_22_ had a weak positive influence on the final size of the cumulative forwarding quantity and had no obvious influence on the propagation times and velocities. In contrast, the average exposure rate *β*_21_ and the extensive exposure attractiveness index *m*_21_ had no significant effect on large interval delay in transmission based on forwarding. All the aforementioned key parameters had no significant effect on the outbreak time, climax time, and duration of the long-delayed cross-information transmission based on forwarding, which was also consistent with the results of the partial rank correlation coefficients.

### Influencing Factors Analytics: Information Release and Dissemination for the STI DT-SFI Model

**Figure 15:**
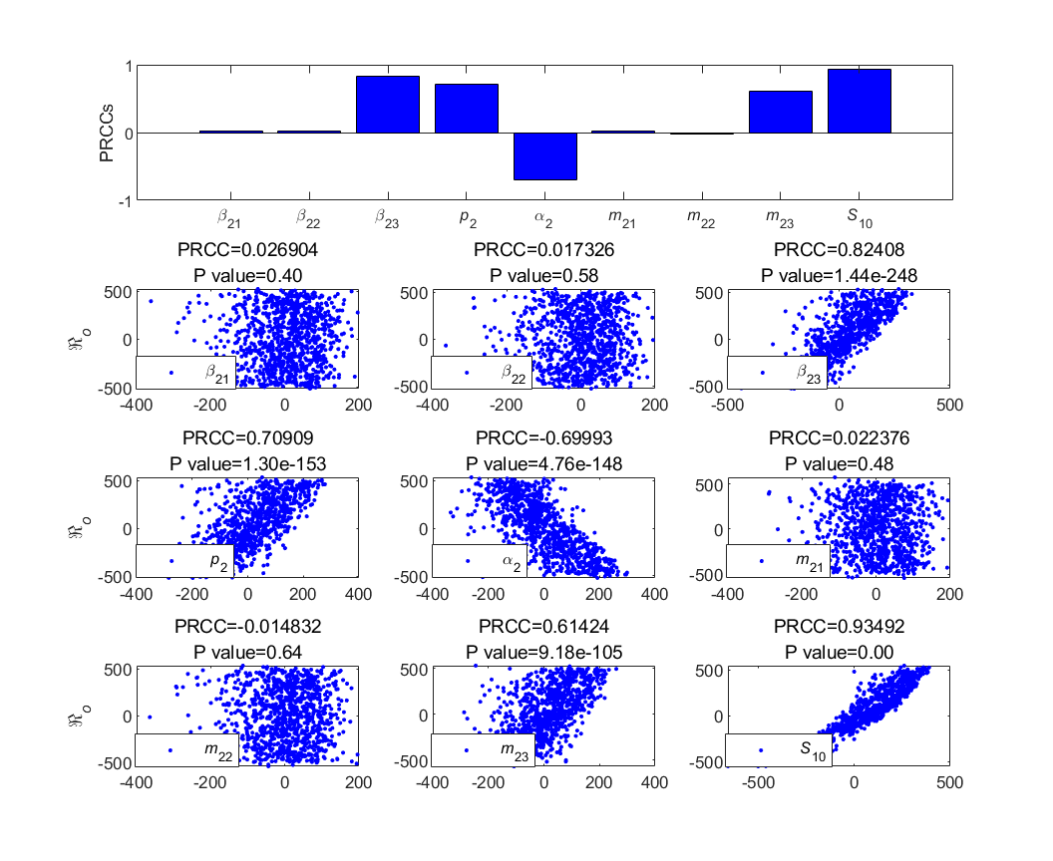
PRCC results and PRCC scatterplots with indexes 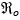 for different parameters of the newly posted information in the short interval delay in transmission–susceptible-forwarding-immune model. PRCC: partial rank correlation coefficient.

**Figure 16:**
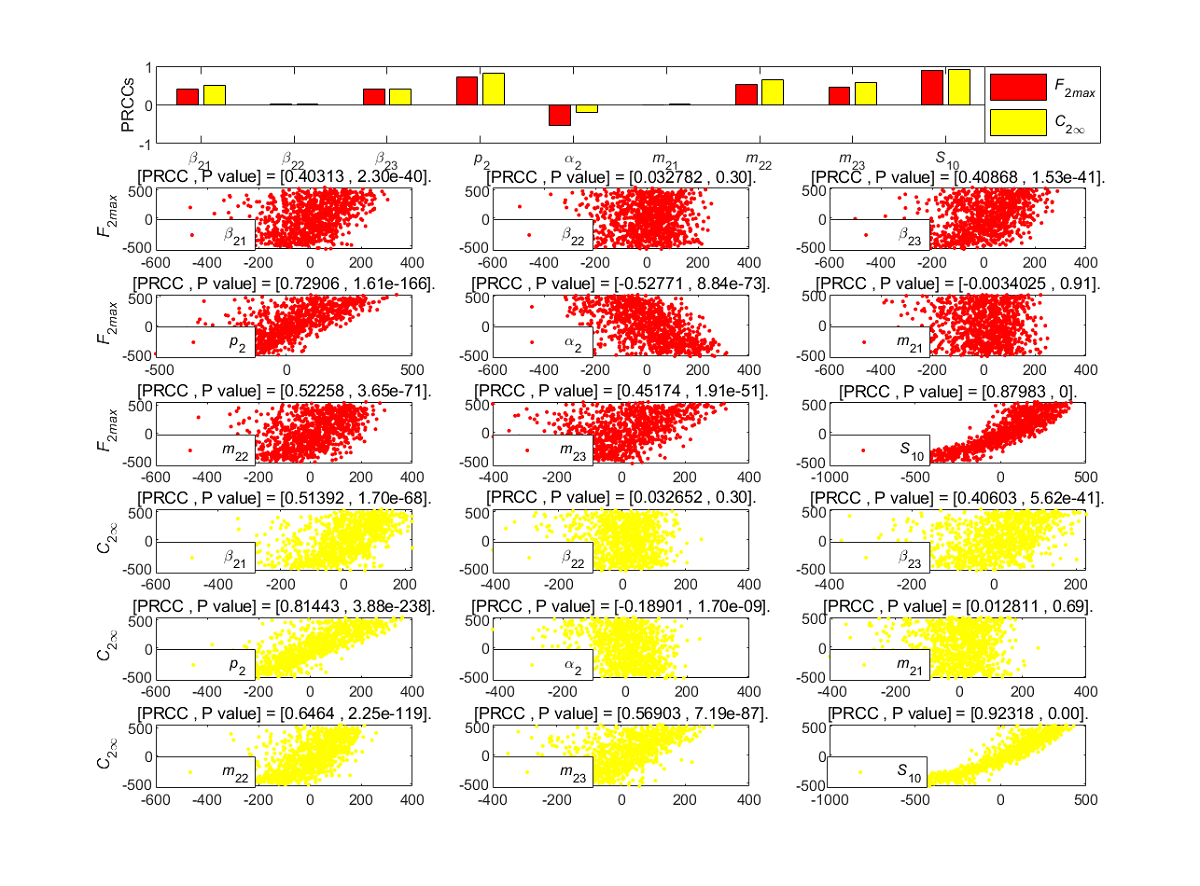
PRCC results and PRCC scatterplots with indexes *F*_2*max*_ and *C*_2∞_ for different parameters of the newly posted information in the short interval delay in transmission susceptible-forwarding-immune model. PRCC: partial rank correlation coefficient.

**Figure 17:**
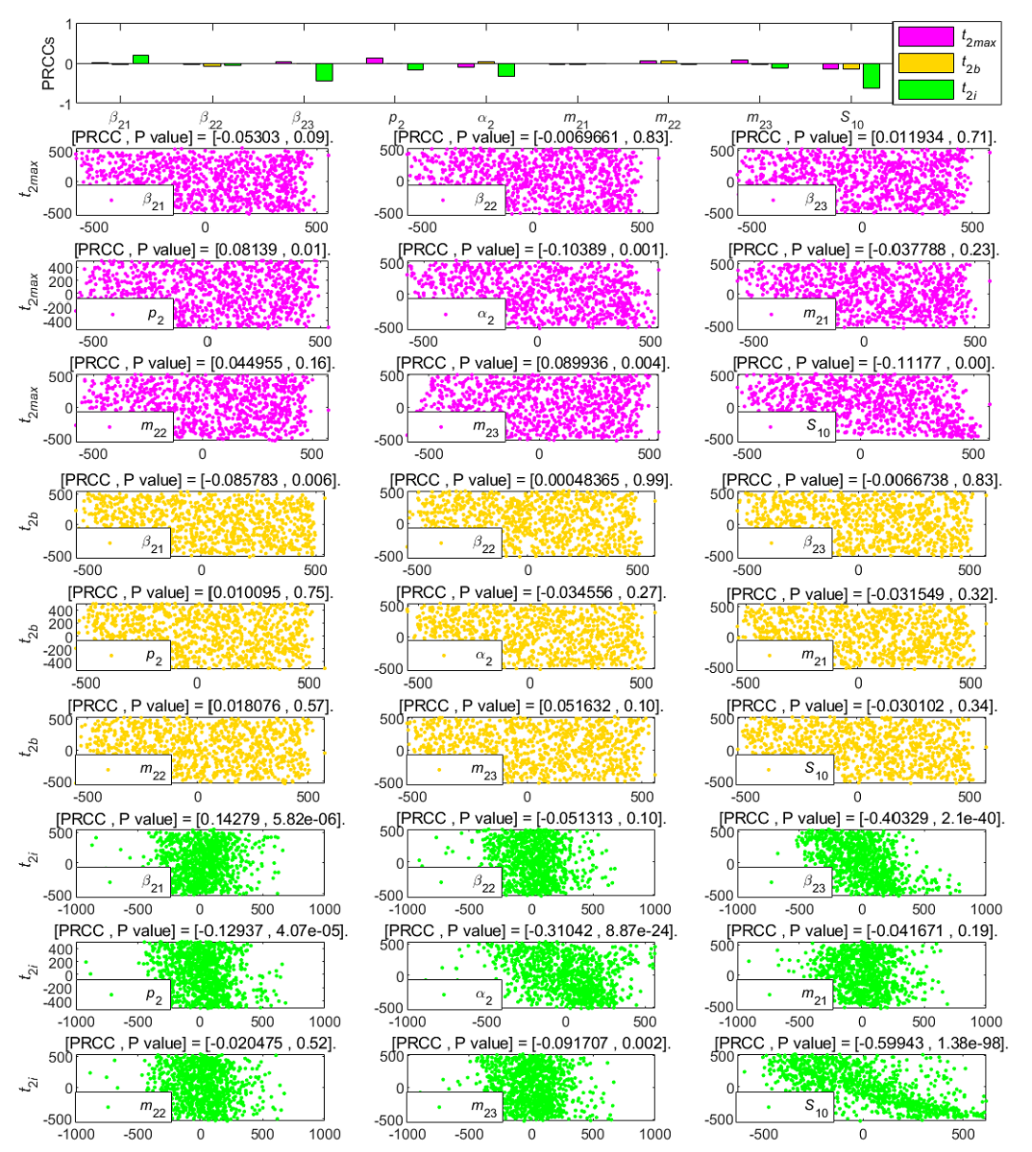
PRCC results and PRCC scatterplots with indexes *t*_2*max*_, *t*_2*b*_, and *t*_2*i*_ for different parameters of the newly posted information in the short interval delay in transmission susceptible-forwarding-immune model. PRCC: partial rank correlation coefficient.

**Figure 18:**
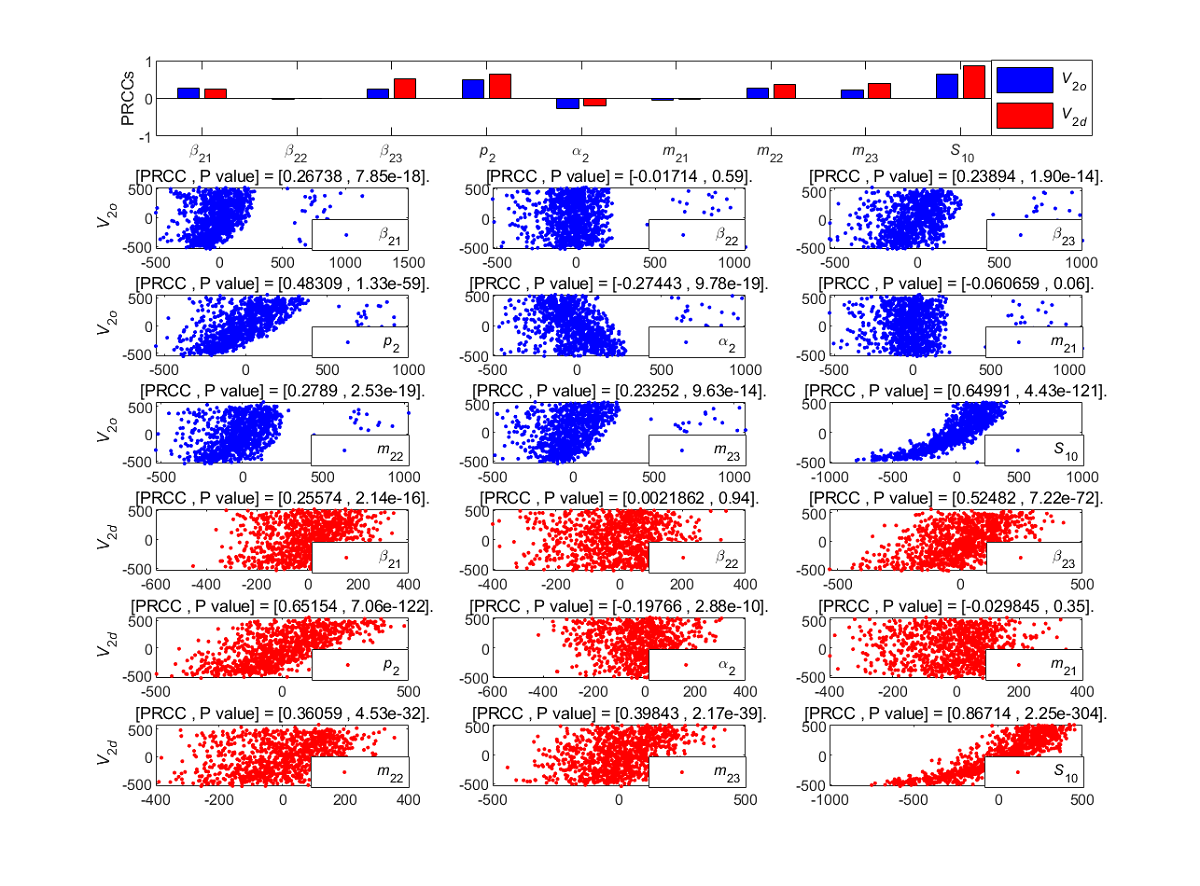
PRCC results and PRCC scatterplots with indexes *V*_2*o*_ and *V*_2*d*_ for different parameters of the newly posted information in the short interval delay in transmission susceptible-forwarding-immune model. PRCC: partial rank correlation coefficient.

To further analyze the impact of different parameters in the STI DT-SFI model for the cross-propagation dynamics, we performed partial rank correlation coefficients to analyze the relationship between the influence and the range of variation of parameters on the indexes. Figures 15-18 give the partial rank correlation coefficient results and partial rank correlation coefficient scatterplots with indexes 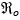, *F*_2_*_max_*, *C*_2∞_, *t*_2_*_b_*, *t*_2_*_i_*, *t*_2_*_max_*, *V*_2_*_o_*, and *V*_2_*_d_* with nine parameters (*β*_21_, *β*_22_, *β*_23_, *p*_2_, *α*_2_, *m*_21_, *m*_22_, *m*_23_, and *S*_10_) of the newly posted information in the STI DT-SFI model, respectively.

The average exposure rate *β*_23_, the unexposure attractiveness index *m*_23_, the forwarding probability *p*_2_, and the initial value *S*_10_ were strong contributions to the public opinion reproduction ratio 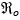 positively, and the average immune rate *α*_2_ had a strong negative effect on it, as shown in Figure 15. The positive correlation effects of parameters *β*_21_, *β*_22_, *m*_21_, and *m*_22_ were relatively weak. In general, strategies that can affect the parameters *β*_23_, *m*_23_, *p*_2_, and the initial value *S*_10_ to increase or the parameter *α*_2_ to decrease can increase the initial propagation capacity of the newly posted information. On the other hand, we can decrease the parameters *β*_23_, *m*_23_, *p*_2_, and the initial value *S*_10_ to reduce the initial propagation ability of the new information.

The average contact rate of *β*_21_, *β*_22_, the forwarding probability *p*_2_, the mild exposure attractiveness index *m*_22_, the unexposure attractiveness index *m*_23_, and the initial value *S*_10_ of susceptible individuals had strong positive impacts on the high peak *F*_2_*_max_* and the final size *C*_2∞_, as shown in Figure 16. In contrast, *S*_10_ played a major role, and the impact of *β*_22_ and *m*_21_ were less significant. The aforementioend results show that individuals who have been exposed to but have not forwarded the posted information are more sensitive to the new information with mild exposure attractiveness due to the understanding of the former information. In addition, the average immune rate *α*_2_ had a strong negative effect on the *F*_2_*_max_*.

Figure 17 indicates that the influence of each parameter on *t*_2_*_max_* and *t*_2_*_b_* were not obvious, and the parameters *β*_23_, *α*_2_, and the initial value *S*_10_ had negative effects on the duration *t*_2_*_i_*, and the *β*_21_ had a weak positive effect on it. This means that the average contact rate at which users in the susceptible state can contact the second information is the most important factor affecting the duration *t*_2_*_i_* of delay in transmission. The smaller the average contact rate is, the longer the duration of new information transmission will be within a certain range, slowing down the development of information transmission.

Figure 18 shows the partial rank correlation coefficients results of the outbreak velocity *V*_2*o*_ and the declining velocity *V*_2*d*_ of the STI DT-SFI model based on forwarding under multiparameter changes. From the results, the average exposure rate *β*_21_, *β*_23_, the forwarding probability *p*_2_, the mild exposure attractiveness index *m*_22_, the unexposure attraction index *m*_23_, and the initial value *S*_10_ of susceptible individuals make strong positive contributions on *V*_2_*_o_* and *V*_2*d*_. The average exposure rate *β*_22_ and the extensive exposure attractiveness index *m*_21_ had no significant effect on the velocities. That is to say, the outbreak velocity *V*_2*o*_ and the declining velocity *V*_2*d*_ can be increased with the increase of the parameters *β*_21_, *β*_23_, *p*_2_, *m*_22_, *m*_23_, and the initial value *S*_10_. On the contrary, if the parameters decrease, the propagation velocities will slow down.

**Figure 19:**
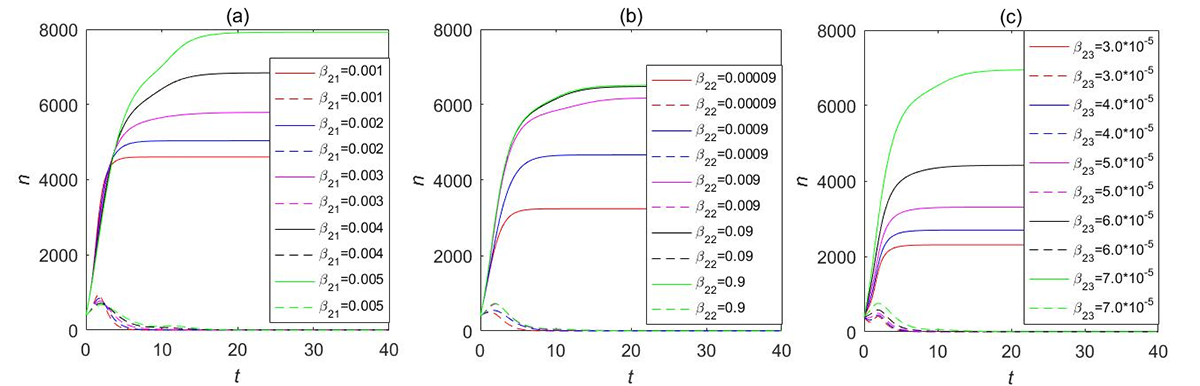
The influence of the average exposure rates on the instantaneous forwarding population *F*_2_(*t*) and the cumulative forwarding population *C*_2_(*t*) in the short interval delay in transmission susceptible-forwarding-immune model.

**Figure 20:**
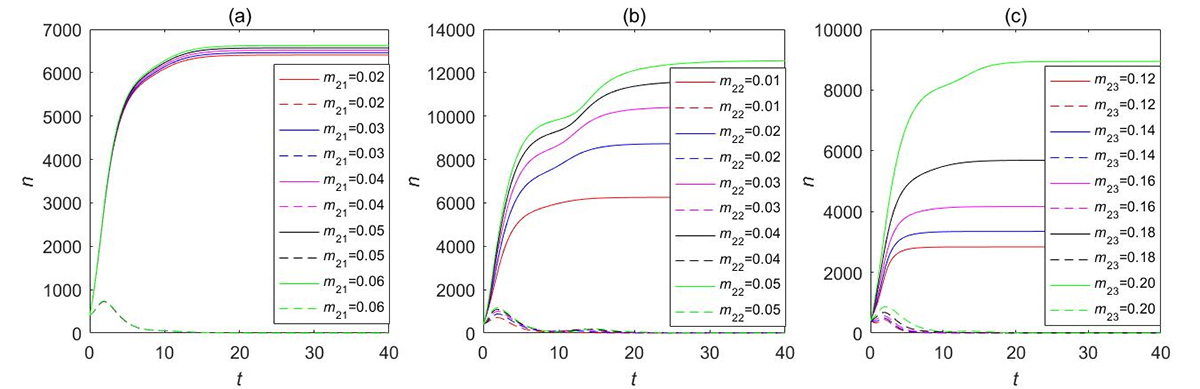
The influence of the attractiveness parameters on the instantaneous forwarding population *F*_2_(*t*) and the cumulative forwarding population *C*_2_(*t*) in the short interval delay in transmission susceptible-forwarding-immune model.

Here, we also took into consideration the influence of the average exposure rate and attractiveness parameters on the instantaneous forwarding population *F*_2_(*t*) and the cumulative forwarding population *C*_2_(*t*) of the STI DT-SFI model as shown in Figures 19 and 20, respectively. The comparative analysis shows that the larger the average contact rate and attractiveness indexes are, the larger the instantaneous forwarding quantity and the cumulative forwarding quantity are. The final size is also affected; the average exposure rate of *β*_21_, *β*_23_, the mild exposure attractiveness index *m*_22_, and the unexposure attractiveness index *m*_23_ are the main influencing factors of the STI DT-SFI model, and they can play a significant role in the final size of the newly posted information within a certain range. So priority must be placed on controlling these parameters. In addition, the extensive exposure attractiveness index *m*_21_ has only a small magnitude of effects, while the effect of parameter *β*_22_ is significant and has a relatively obvious impact. The impact of each parameter on the outbreak timing and increasing and declining velocities is negligible, which is consistent with the results of the partial rank correlation coefficients.

## Discussion

### Principal Findings

**Figure 21:**
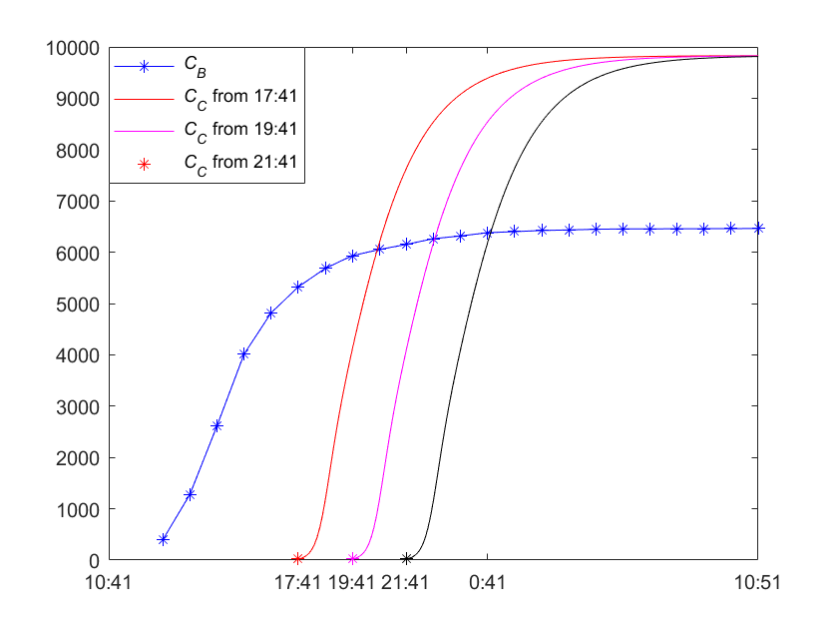
An illustration of a public opinion dissemination process with newly posted information posted with different time lags but during the quasi–steady-state period of the posted information.

Figure 21 shows the trend of the cumulative forwarding users of information B and information C when the newly posted information is posted during the steady-state period of the posted information in Tables 2 and 3. The time lag with which the newly posted information is posted has a significant impact on the process of public opinion dissemination and the final size of the cumulative number of forwarding users. If the newly posted information is posted during the quasi–steady-state period of opinion propagation, then the earlier the newly posted information is posted, the earlier the cumulative forwarding users will peak, though the final size of the cumulative forwarding users will be close to each other. This, in conjunction with our parameter sensitivity analysis results, shows that for the LTI DT-SFI situation, the unexposure attractiveness index *m*_23_ and the average exposure rate *β*_23_ are the key elements to promote the cross-propagation and that, once reaching the quasi–steady state, the timing of posting the new information has an insignificant impact on the final size of forwarding users.

**Figure 22:**
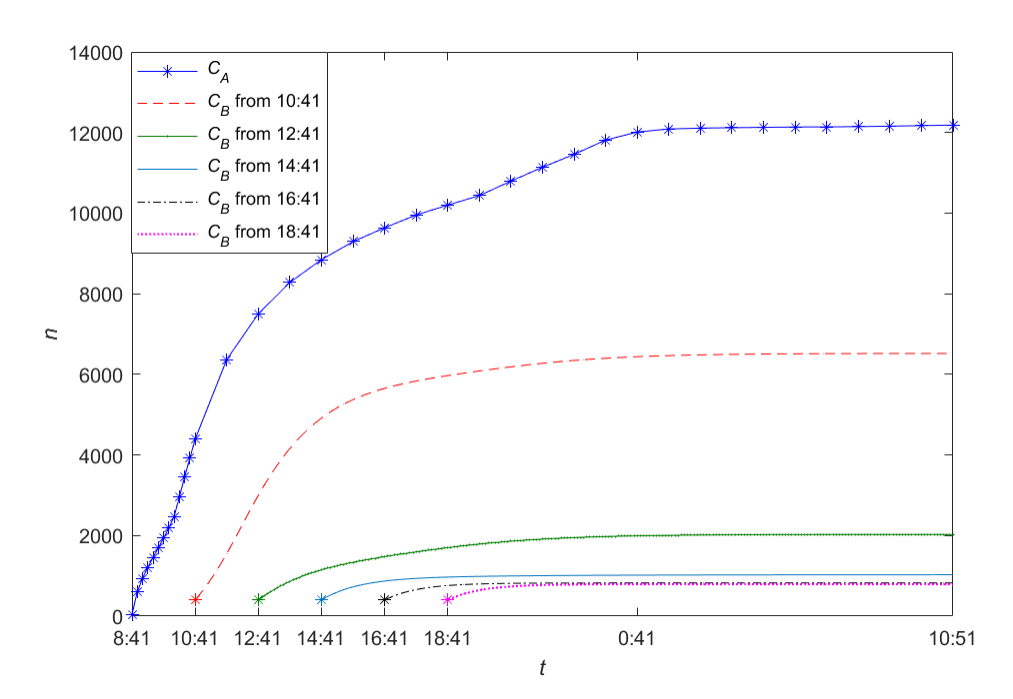
An illustration of a public opinion dissemination process with newly posted information posted with different time lags but during the quasi–steady-state period of the posted information.

In contrast, Figure 22 shows the trend of the cumulative forwarding users of information A and information B when the newly posted information is posted during the outbreak period of the posted information in Tables 1 and 2. The time lag with which the newly posted information is posted has a noticeable impact on both the dynamic process of public opinion dissemination and the final size of the cumulative number of forwarding users. If the newly posted information can be posted during the outbreak period of the old information, then the earlier the new information is posted, the greater the cumulative forwarding population and the final size of the cumulative forwarding users will be. This, combined with our parameter sensitivity analysis results, shows that, in the STI DT-SFI case, the mild exposure attractiveness index *m*_22_, the average exposure rates *β*_21_ and *β*_23_, and the unexposure attractiveness index *m*_23_ can all directly influence the interaction between information posted sequentially to increase the “heat” (popularity) of the newly posted information.

Our model-based analysis recommends strategies on how different parameters should be adjusted to achieve the best information dissemination outcomes. For two pieces of relevant information separated by a relative long posting lag, strategies to increase the average exposure rate *β*_23_ and the unexposure attractiveness index *m*_23_ are recommendations. These strategies can be achieved if opinion leaders with a large number of followers can participate in the information copropagation. On the contrary, reducing the public’s attention to a new piece of information can be achieved by efforts in delaying the posting of the new information or by effectively reducing the potential correlation between the two pieces of information (reducing values of the correlation parameters *β*_21_, *β*_22_, *m*_21_, and *m*_22_). Additionally, if our goal is for the final size of the cumulative forwarding users of the new information to not be impacted by the relevant information already posted online, the new information should be posted during the quasi–steady-state period of the posted information.

For two pieces of information with a short interval between posting, we recommend developing strategies to alter the interaction between the information for effectively managing the information transmission indexes we introduced. If we aim to make the new information outbreak faster with a large peak value of forwarding, we should increase the average exposure rate *β*_21_ and mild exposure attractiveness index *m*_22_ by persuading the original post owner to post or forward the information earlier during the outbreak period of the posted information, when the posted information has obtained certain public attention, and increase the relevance and attraction of the newly posted information to the forwarding users or immune users of the posted information. Alternatively, we should persuade some opinion leaders to forward the new information along with their insights to *β*_23_ and *m*_23_.

### Conclusions

Relevant information is often posted sequentially in fast-evolving public health events such as the COVID-19 pandemic. Consequently, modeling the impact of delay in cross-transmission or copropagation is substantial to identifying the best strategies to communicate key public messages through social media. In this study, we proposed and examined two classes of models, the LTI DT-SFI dynamics model and the STI DT-SFI dynamics model based on the forwarding users in Weibos, and we parametrized our models using real data related to the COVID-19 pandemic in the Chinese Sina Microblog. Our goal is to use these parametrized models to understand the influence of different time lags in the information posting on the copropagation of related information in the microblog.

Our model formulation focused on the transmission mechanism of information in the social network, where a new Weibo may be posted in different phases—outbreak phase or quasi–steady-state phase—of some relevant Weibo already posted. Our goal is to examine the impact of post timing in relation to the old information, the new information on its peak value, and the final size of forwarding users. As we have shown, this impact depends on the correlation of the old and new information, and on the phase of the old information transmission when the new information is posted. We hope that our DT-SFI dynamics models fill in some theoretical gap about optimizing information posting strategies to maximize communication efforts to deliver key public health messages to the public for better outcomes of public health emergency management.

## Abbreviations

DT-SFI: delay in transmission susceptible-forwarding-immune
LS: least squares
LTI DT-SFI: large interval delay in transmission susceptible-forwarding-immune
SIR: susceptible-infected-recovered
STI DT-SFI: short interval delay in transmission susceptible-forwarding-immune
